# Combining Small-Vertebrate, Marine and Stable-Isotope Data to Reconstruct Past Environments

**DOI:** 10.1038/srep14219

**Published:** 2015-09-22

**Authors:** Juan Rofes, Naroa Garcia-Ibaibarriaga, Mikel Aguirre, Blanca Martínez-García, Luis Ortega, María Cruz Zuluaga, Salvador Bailon, Ainhoa Alonso-Olazabal, Jone Castaños, Xabier Murelaga

**Affiliations:** 1 Archéozoologie, Archéobotanique : Sociétés, pratiques et environnements (UMR 7209), Sorbonne Universités, Muséum national d’Histoire naturelle, CNRS, CP56, 55 rue Buffon, 75005 Paris, France; 2 UPV-EHU, Facultad de Ciencia y Tecnología, Departamento de Estratigrafía y Paleontología, Apartado 644, E-48080 Bilbao, Spain; 3 UPV-EHU, Facultad de Letras, Departamento de Geografía, Prehistoria y Arqueología, c/Tomás y Valiente s/n, 01006 Vitoria-Gasteiz, Spain; 4 UNED, CA Bergara, San Martin Agirre Plaza 4, E-20570 Bergara, Spain; 5 UPV-EHU, Facultad de Ciencia y Tecnología, Departamento de Mineralogía y Petrología, Apartado 644, E-48080 Bilbao, Spain; 6 UMR 7209-UMR 7194, CNRS Départament Ecologie et Gestion de la Biodiversité (EGB), Sorbonne Universités, Muséum national d’Histoire naturelle, CNRS, CP55, 55 rue Buffon, 75005 Paris, France

## Abstract

Three very different records are combined here to reconstruct the evolution of environments in the Cantabrian Region during the Upper Pleistocene, covering **~**35.000 years. Two of these records come from Antoliñako Koba (Bizkaia, Spain), an exceptional prehistoric deposit comprising 9 chrono-cultural units (Aurignacian to Epipaleolithic). The palaeoecological signal of small-vertebrate communities and red deer stable-isotope data (δ^13^C and δ^15^N) from this mainland site are contrasted to marine microfaunal evidence (planktonic and benthic foraminifers, ostracods and δ^18^O data) gathered at the southern Bay of Biscay. Many radiocarbon dates for the Antoliña’s sequence, made it possible to compare the different proxies among them and with other well-known North-Atlantic records. Cooling and warming events regionally recorded, mostly coincide with the climatic evolution of the Upper Pleistocene in the north hemisphere.

The reconstruction of past environments has been addressed from many disciplines and perspectives in the past, being fundamentally concerned with two things: a well-defined and reasonably complete environmental record and an adequate chronological framework. A holistic effort in this sense, is to be found at the recently published results of the INTIMATE project^1^. An extensive compilation of climatic and palaeoenvironmental reconstructions of the 60—8 ka period in the North Atlantic Region is presented therein, with authors using diverse records, as the Greenland ice-cores, tree-rings, pollen, tephrochronology, speleothems, and marine proxies, among others. Independently, for the first time, we combine here radiometric dating with three very different proxies, i.e., small vertebrates, stable isotopes of herbivores, and marine record (ostracods and foraminifers), commonly used each one of them separately^2^,^3^,^4^, to reconstruct the evolution of landscapes and environments in the Cantabrian Region (Fig. 1) during the Upper Pleistocene, covering a time span of ~35.000 years.

The first proxy is based on small vertebrates (Fig. 2). Digestion sub-products of birds of prey (i.e., rejection pellets) and small carnivores are the main sources for small-vertebrate deposition in archaeological sites^5^, being caves and rock shelters particularly propitious places for those animals to nest at the entrances, or to build burrows inside, respectively^3^,^6^,^7^. Unlike large mammal remains (many of them product of human selection), small-vertebrate accumulations reasonably well reflect local biocenosis, despite unavoidable filters due to specific predators^5^. Moreover, small vertebrates are particularly sensitive to climatic and habitat changes, and their shifts along time in terms of taxa and number of specimens can be successfully used for the reconstruction of past environments^3^,^6^,^7^,^8^,^9^,^10^,^11^.

Another proxy is given by the analysis of the stable-isotope data from bone collagen of herbivores. Variability in climate and local environment determines food source availability, and different food sources have particular stable carbon and nitrogen isotope values. Since the food isotopic signal is reflected in bone collagen, this can be used as a proxy to infer palaeodietary and palaeoclimatic variations^12^,^13^. The stable carbon isotope ratio (δ^13^C) of herbivore tissue is related to factors such as the environment, the photosynthetic pathways of consumed plant matter, humidity, water availability, salinity, and partial atmospheric pCO_2_^14^,^15^. For instance, plants in woody landscapes appear to lead to δ^13^C depletion compared to plants of open habitats (the so-called ‘canopy effect’^16^,^17^). By contrast, nitrogen isotope ratios (δ^15^N) preserved in animal tissues are related to factors such as diet, climate, and water availability^18^,^19^,^20^,^21^. In addition, the δ^15^N of herbivores’ bone collagen may reflect processes of soil formation, especially in environments with influence of permafrost^19^,^20^,^22^. During cold periods, the δ^15^N in the collagen of herbivores shows lower values, and thereafter, from a diachronic standpoint, an increase of δ^15^N values over time may be due to temperature rising parallel to higher organic activity in soils^19^,^20^,^22^,^23^. As plant δ^13^C and δ^15^N signals reflect environmental parameters, and isotope variations occur during periods of great climatic change, variations in collagen isotope values reflect the effects that the environment may have had on fauna^19^,^20^,^21^,^22^,^23^,^24^.

The marine record, represented here by planktonic and benthic foraminifers and ostracods gathered at the southern Bay of Biscay (Fig. 3), not so far from Antoliñako Koba site (see Fig. 1 and Methods), has been extensively used for palaeoenvironmental reconstructions in the Quaternary period^4^,^25^,^26^,^27^,^28^. The planktonic foraminifer *Neogloquadrina pachyderma* sinistral (sin), in particular, is a polar species that appears in percentages of >90% to the north of the North Atlantic Polar Front^29^, being its abundance a useful proxy to identify the meridional migration of the Polar Front during the Quaternary. In the western Iberian Margin, high quantities of this taxon are connected to colder episodes of the last glacial period^30^. Hence, peaks of this species can be reasonably correlated with cold climate events (i.e., Heinrich Events and Greenland Stadials). The term “Northern guests” is referred only to ostracod taxa currently living outside the Mediterranean Sea (at northern latitudes), and which arrive to the Mediterranean during “cold” Quaternary climatic episodes^31^. Following this criteria, here we consider as “Northern guests” such ostracod species that do not live today in the Bay of Biscay (having a circumpolar distribution, i.e., “cold-water” proxies), and which enter into the Basque shelf only during some cold climatic events, namely *Acanthocythereis dunelmensis*, *Cytheropteron testudo* and “*Trachyleberis*” sp.

The stable oxygen isotope ratio (δ^18^O) is one of the most extensively used tools in palaeoclimatology and palaeoceanography. In marine sediments, the δ^18^O values of planktonic and benthic foraminifer shells are used as proxies to reconstruct variations in salinity, temperature and isotopic composition of shallow (planktonic) and deep (benthic) sea water, to analyze fluctuations in global ice volume, to identify changes in oceanic water masses, and to construct a high resolution time scale during the Quaternary^32^,^33^. The δ^18^O ratio of many planktonic and benthic foraminifer species, such as *Globigerina bulloides* and *Cibicides* spp., has been considered to be in equilibrium with the δ^18^O signal of surface and bottom waters, or to have a constant offset, what allows to identify changes in water masses properties and establish a consistent stratigraphy of marine sedimentary cores of the North Atlantic Ocean^34^,^35^. Therefore, here we use the oxygen isotopic signal δ^18^O measured in *Lobatula lobatula* (benthic foraminifer) and *G. bulloides* (planktonic foraminifer) to detect changes on the characteristics of, respectively, bottom and surface water masses in the Basque shelf during the late Quaternary.

Antoliñako Koba (AK) (Gautegiz-Arteaga, Bizkaia, Spain: Fig. 1a) is an exceptional archaeo-palaeontological karstic deposit with a long Upper Pleistocene sequence, comprising nine chrono-cultural units: Aurignacian, evolved Aurignacian, Gravettian, Upper Solutrean, late Lower Magdalenian, Upper Magdalenian, Azilian, and ancient Epipaleolithic^36^,^37^,^38^. There are also scarce pre-Aurignacian evidences of human presence in an underlying still undated layer^36^. The chronology of AK roughly goes from nearly 44 to 9 ka cal BP. Details of the stratigraphy, lithic industries, portable art, and contextualization of the site in the regional prehistory are to be found elsewhere^36^,^37^,^38^,^39^,^40^. The good preservation of the materials from AK allowed us to obtain both the small-vertebrate collection and the red deer (*Cervus elaphus*) bone samples, the latter used for stable isotope analysis (see Methods). Pollen cores were also taken from the entire sequence. Unfortunately they revealed to be sterile in all cases (María José Iriarte, pers. comm.).

Many radiocarbon dates obtained for the AK’s sequence (see Table 1 and Fig. 4, 1^st^ and 2^nd^ columns), made it possible to correlate the land and marine records between them, and then with sedimentological and palynological episodes of the Cantabrian Region^41^,^42^, with the variations of the eustatic sea level^43^,^44^, and with a δ^18^O curve from a deep ice-core of north Greenland (NGRIP^45^). Two main objectives are pursued in this study: 1) contrast the mainland and marine records of the same region, filling the gaps that commonly exist along the mainland sequences with the usually more complete marine record; and 2) obtain a continuous palaeoenvironmental reconstruction for the 44–9 ka cal BP period at the Cantabrian Region by correlating and cross-checking several proxies (small vertebrates, marine record and stable isotopes of herbivores), correlating those proxies in turn with well-known North-Atlantic records.

## Results

### Small vertebrates

The small-vertebrate assemblage from AK comprises 28 taxa: four soricids (*Sorex* cf. *coronatus*, *S. minutus*, *Neomys* sp., and *Crocidura russula*); one talpid (*Talpa* sp.); one sciurid (*Marmota marmota*); two glirids (*Glis glis* and *Eliomys quercinus*); two murids (*Apodemus sylvaticus* and *A. flavicollis*); seven cricetids (*Arvicola amphibius*, *Chionomys nivalis*, *Pliomys lenki*, *Microtus* (*Terricola*) *lusitanicus*, *M.* (*Alexandromys*) *oeconomus*, *M.* (*Microtus*) *arvalis*, and *M.* (*M.*) *agrestis*); five amphibians (*Alytes obstetricans*, *Bufo bufo*, *B. calamita*, *Rana* gr. *temporaria-iberica*, and *Triturus* sp.); and six reptiles (*Anguis fragilis*, *Chalcides striatus*, *Coronella girondica*, cf. *Natrix*, *Vipera* sp., and Lacertidae indet.).

Figure 4 reconstructs the AK habitat distribution based on changes in the small-mammal (7^th^ column) and amphibian and reptile (8^th^ column) communities over time, respectively. The frequencies of the different taxa (expressed in NISP and MNI) and their habitat affinities are given in supplementary Tables 1 and 2. The species were ordered by stratigraphic levels covering the Aurignacian to ancient Epipaleolithic periods (Sm-P to Lanc), plus some pre-Aurignacian layers without a specific chrono-cultural assignation (Sm to B-Am). There are certain hiatuses and erosive episodes along the sequence shown in Fig. 4.

First to be noticed is the pre-eminence of forests at the bottom of the sequence. During pre-Aurignacian times, woodlands were important in the vicinity of the cave according to both the amphibian and reptile (AR) record (Level Sm) and the small-mammal (SM) record (Level Lsm-P). For the first clearly defined archaeological level (Sm-P), and since slightly before, the SM record documents two peaks of forest, the highest of the sequence, with a moderate presence of open landscapes as well. There is a notorious peak of rocky habitats at the top of Sj/P. The level called lower Lmbk/Smk, assigned to the evolved Aurignacian, is characterized by an equilibrium between woodland and meadows according to the SM, and by a peak of open landscapes according to the AR. There is no contradiction but alternation in this case, as shown in Fig. 4.

Some degree of contradiction between the SM and AR records is present in the Gravettian layers. While the SM show a nearly similar representation of forests and meadows, the AR exhibit two peaks of meadow and one of woodland, with more presence of water component than in the SM. This discrepancy may be a bias due to the lower NISP of AR respect to SM in these particular levels (compare upper Lmbk/Smbk + Lab/Sab in supplementary Tables 1 and 2). After the second hiatus of the sequence, during Upper Solutrean times, and according to the SM, there are two peaks of forest at levels Lmc and Lmb, respectively, and a significant increase of rocky habitats (in detriment of woodland and meadows) in Lmb, immediately before the second peak of forest. The AR record supports the two forest peaks of the SM, being them considerably higher in AR.

The late Lower Magdalenian (lower Lgc) shows a predominance of woodland over open landscapes, especially in the SM. This tendency continues after a long hiatus. During Azilian times (upper Lgc) there are peaks of meadows in detriment of water habitats both in the SM and AR. The forest component remains high, but more according to the SM. Finally, towards the Ancient Epipaleolithic (represented by Level Lanc), we observe a reduction of woodland and open territory in favor of waters at the SM column, but an increase of meadows in the AR, in detriment of both forest and water habitats. The rocky areas are moderately represented since the Azilian to the top of the sequence, but only at the AR column.

### Stable carbon and nitrogen isotopes

The results of the carbon (C) and nitrogen (N) isotope analysis for the *C. elaphus* bone samples of AK are displayed in columns 9^th^ and 10^th^ of Fig. 4, respectively. The variation in red deer collagen δ^13^C values along the sequence is due to consumption of different types of plants. *Cervus elaphus* is classified as an intermediate feeder, with a mixed diet between grazing and browsing^46^,^47^. Red deer δ^13^C values could be attributed to graminoid and shrubbery feeding, particular to semi-open grasslands^18^,^19^,^20^. The δ^13^C values of AK specimens show a decreasing trend from the Upper Solutrean to the Azilian (~23–12 ka cal BP), ranging from δ^13^C −20.3 to −21.0‰ (supplementary Table 3 and supplementary Fig. 1a). This variation suggests a progressive increase in forest cover^16^. From the Aurignacian to the Gravettian (~36–30 ka cal BP) an opposite trend is recorded, probably reflecting a decrease of woodlands along this period.

Regarding the nitrogen, we observe an increase in δ^15^N values from the Upper Solutrean to the Azilian, mean values varying between δ^15^N 2.8 and 4.4‰ (supplementary Table 3 and supplementary Fig. 1b). This increase could be the result of a progressive rise in temperature^19^,^20^,^22^,^23^ (see Introduction). From the Aurignacian to the Gravettian the trend is ambiguous. During the Gravettian (upper Lmbk/Smbk + Lab/Sab), δ^15^N values exhibit two sets of isolated values: one with high values (δ^15^N = 7–8‰) and other with lower values (δ^15^N < 5‰). The presence of these two sets in the red deer of AK could represent populations coming from different territories. If we interpret the higher values as belonging to non-local individuals, and we assume the lower values as valid, then the resulting tendency to a decrease in temperatures would be in agreement with the trend inferred from the δ^13^C (compare supplementary Figs. 1a and 1b).

### Marine record

The palaeoceanographic evolution of the Basque shelf during the late Quaternary, based on microfaunal (% of *N. pachyderma* sin and “Northern guest” ostracod species) and isotopic (δ^18^O on *G. bulloides* [bull] and *L. lobatula* [lob]) signals, is shown in columns 11^th^ and 12^th^ of Fig. 4, reflecting the influence of diverse water masses in this area.

During MIS 3, the high percentage of *N. pachyderma* sin, together with the positive values of δ^18^O_bull_, indicates the influence of cold polar surface waters in the Basque shelf. Regarding the benthic record, the isotopic signal (δ^18^O_lob_) marks the presence of cold bottom waters in the Basque shelf. However, the scarcity of “Northern guest” ostracod species shows the absence of inputs of subpolar bottom waters^4^. The HE 3 (~31.3 ka cal BP) is a remarkable period: the marine record reflects the arrival of both surface and bottom cold subpolar waters into the Basque shelf 4,28.

At 23.9 ka cal BP, Heinrich Event (HE) 2 is detected by an increase in the percentage of *N. pachyderma* sin synchronic to a peak of δ^18^O_bull_, reflecting the arrival of cold polar surface waters^4^,^28^. This event is followed by a decrease in the isotopic signal (~23.2 ka cal BP) that corresponds to the warm event GI-2. The decrease in abundance of *N. pachyderma* sin and δ^18^O_bull_ values during the Last Glacial Maximum (LGM) compared to HE 2, suggests an increase in temperature, a decrease in salinity of surface sea water^48^, and/or homogenisation of the water column^49^. However, the benthic signal reflects an input of cold subpolar bottom waters into the Basque shelf at ~21.3 ka cal BP^4^. This cold water shift affected only bottom waters, and retreated during the rest of the LGM.

At the end of LGM (~20.1 ka cal BP), a new peak of abundance of *N. pachyderma* sin shows the entrance of cold polar surface waters into the Basque shelf. This can be correlated with the retreat and melting of the European ice sheets and glaciers occurred at 20 ka cal BP^50^, which caused a sea-level rise at around 19 ka cal BP (19 ky-melt water pulse; 19 ky-MWP in Fig. 4). From ~18.6 to ~15 ka cal BP, the HE 1 is characterized by the variations in relative abundance of *N. pachyderma* sin, “Northern guests” and δ^18^O_bull_ and δ^18^O_lob_ signals, reflecting the input of both cold surface and bottom water masses into the Basque shelf, coming from the circumpolar area of the North Atlantic Ocean^4^,^28^. These trend can be correlated with the age previously proposed for HE 1 in the Bay of Biscay, i.e., ~18 ka cal BP^51^,^52^,^53^.

The erosional hiatuses observed first at the beginning of MIS 2 (27.7–23.9 ka cal BP) and then at the beginning of the second phase of HE 1 (HE 1b)^52^ (17.1–16.5 ka cal BP), seem to be related to the sea level rise starting at ~17 ka cal BP^54^ that characterized the last Deglaciation (14.7–11.5 ka cal BP, beginning of MIS 1)^4^,^28^. This sea-level rise shows its maximum rates from ~14.6 to ~13.8 ka cal BP, during the melt-water-pulse 1a event (mwp-1a in Fig. 4)^54^. After the end of the Younger Dryas (YD, 11.3 ka cal BP) it comes a period of higher sea-level rates, called mwp-1b^54^ (Fig. 4), being the sea-level approximately −60 m respect to the current level. On the continental shelf of the Armorican margin (French margin, northern part of Capbreton Canyon), similar erosive processes were observed during the last Deglaciation, due to the same phenomenon^55^. In the Basque shelf, this rise, which occurred during the beginning of MIS 1 (last Deglaciation and early Holocene), is accompanied by a warming tendency on both surface and bottom waters, as shown by the marine proxies. The increase in abundance of “Northern guests”^4^,^28^ and δ^18^O_lob_ signal at ~9.4 ka cal BP implies the input of cold subpolar bottom waters that may correspond to the Holocene Cooling Event (HCE) 6^56^.

## Discussion

Many proxies have been previously used to reconstruct past environments (e.g., Greenland ice-cores, tree-rings, pollen, tephrochronology, speleothems, and marine record), and some comprehensive holistic efforts have been recently accomplished^1^, but none of them have directly compared small vertebrates, marine record and stable isotopes of red deer to yield a more complete and accurate panorama.

**Figure 1:**
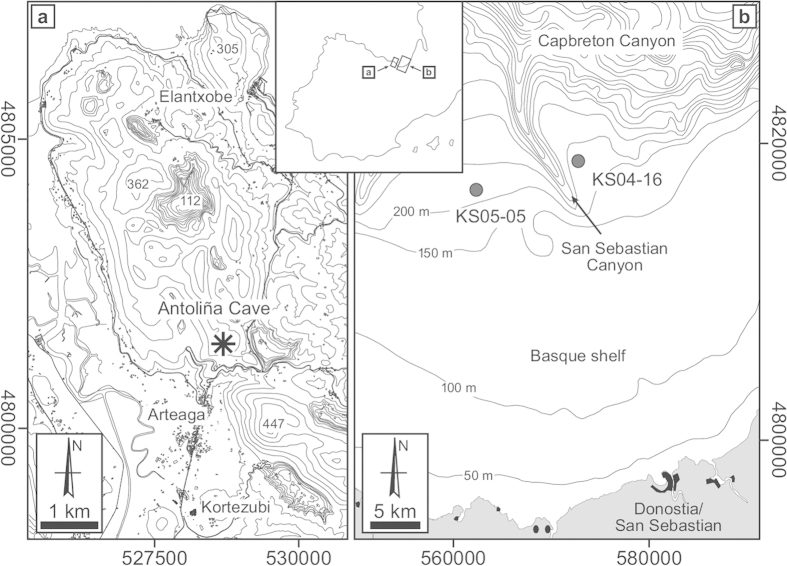
Geographical location. (**a**) Antoliñako Koba site (Gautegiz-Arteaga, Bizkaia, Spain). (**b**) Marine cores taken at the outer Basque shelf (Southern Bay of Biscay). The figure was designed through the combined use of Macromedia Freehand MX, Adobe Illustrator CS6 and Adobe Photoshop Elements 12. Figures 1a and 1b were modified under permission from elsewhere^3^,^28^.

Our study shows how useful it can be to compare the land and marine records, particularly when the samples come from geographically close locations (Fig. 1), what prevents incompatibilities in the sequences. The environmental curves obtained from foraminifers and ostracods of the Southern Bay of Biscay are contemporaneous and complementary to those constructed with the small vertebrates (mammals, amphibians and reptiles) from AK. Some erosive episodes and/or sterile layers along the mainland sequence, provoking gaps in the record, can be successfully fulfilled by the marine record (relative abundances and isotopic signals of certain taxa), as it is the case for the periods ~41.2–37.4 ka cal BP, ~33.6–32 ka cal BP, ~18.2–20.5 ka cal BP, and ~17.6–14.4 ka cal BP. In spite that the marine record is usually more complete than the mainland one^4^,^28^, the long erosive hiatus in the marine cores of the Basque shelf between 27.7 and 23.9 ka cal BP is partially covered by the small-vertebrate sequence.

The different records presented in Fig. 4 can be treated altogether or independently. If taken separately, the small-vertebrate record can be contrasted to other palaeoenvironmental reconstructions performed for the same region, as those of Santimamiñe^3^ (very close to AK), Askondo^11^, El Mirón^6^, El Conde^10^, and Valdavara-1^9^. The mainland stable-isotope evidence can be compared with the recent contributions made for Kiputz IX^2^ and El Mirón^57^; and, finally, the reconstructions done departing from oceanic data (% of species and stable isotopes of foraminifers) can be contrasted to some others obtained for the southern Bay of Biscay^51^,^53^.

If used altogether, it is interesting to have a deeper look into the periods of the sequence where it is possible to contrast the results of the three different records (small vertebrates, marine and stable isotopes) for the Cantabrian Region, and to compare them in turn with some other well-known North-Atlantic records (sedimentological, pollen, eustatic sea-level and NGRIP) to get a wider and more complex view. During the evolved Aurignacian period (~35–33.6 ka cal BP), for instance, small vertebrates show a combined (i.e., mammals plus amphibians and reptiles) scenario of a certain equilibrium between woodland and meadows, which coincides with the intermediate values of the δ^13^C and δ^15^N (supplementary Table 3 and supplementary Fig. 1), meaning moderately forested landscapes and mild temperatures, respectively. The percentages of *N. pachyderma* sin and the slightly positive tendency of δ^18^O_bull_ values indicate moderate influence of polar surface waters in the Basque shelf, its effects in the general environment being thereafter also moderate.

The HE 3 (~31.3–29.8 ka cal BP) in the Basque shelf is clearly shown by the increase of *N. pachyderma* sin, “Northern guests” and the values of δ^18^O_bull_ and δ^18^O_lob_, which indicate the arrival of both surface and bottom subpolar waters, and its consequent influence in the general climate. This corresponds to a trend of deforestation in mainland, as shown by the higher values of δ^13^C respect to the Aurignacian, and a decrease in temperatures if we consider the lower values of δ^15^N as valid (see Results). The combined panorama reflected by the small vertebrates coincides with the δ^13^C values in showing a more open scenario, especially notorious in the amphibian and reptile record. The partial coincidence with the Kesselt pollen phase should not be considered, as the chronology of this warm episode (likely more related to GI-5) and others has been brought into question^42^. At 23.2 ka cal BP there was a remarkable decrease of the *G. bulloides* isotopic signal. At the same time, a woodland peak is detected in the small-mammal record, which roughly coincides with the GI-2 warm event. The peak of rocky habitats at 21.3 ka cal BP, during the LGM, could be related to the high values of δ^13^C (deforestation) and the low values of δ^15^N (fall in temperature). In the oceanic front, high ratios of the planktonic and benthic isotopic signals reflect inputs of cold superficial and bottom waters. There is a peak of “Northern guests” at the same time. In sedimentological and palynological terms, the rocky-habitat peak coincides with the transition between the Cantabrian phases I (Oldest Dryas) and II (Lascaux)^41^, being the former especially cold and humid.

During the occupation of AK by Lower Magdalenian people, a trend of progressive forestation of landscapes can be inferred from both the small-vertebrate record and the δ^13^C values, the δ^15^N ratio showing a parallel, not surprising, rise in temperature respect to previous Solutrean times. The arrival of both bottom and surface cold water masses to the Basque shelf continues over this period, but there is a drop in quantities of *N. pachyderma* sin and negative trends of δ^18^O_bull_ and δ^18^O_lob_ at the uppermost part. These curves nicely fit with phase V of the Cantabrian Region^41^, in the sedimentological front. The Azilian chrono-cultural period roughly coincides with the YD. This is a time of peaks of woodland in the small-vertebrate record, which correspond to forestation after the δ^13^C signal, and to ascending temperatures after δ^15^N values. Numbers of *N. pachyderma* sin and δ^18^O_bull_ values diminish accordingly. The benthic signal, however, shows inputs of subpolar bottom waters towards the half of this period. The palynological (Dryas III) and sedimentological (Phase IX) data reflects mild conditions^41^. This trend of progressive increase in woodland and rise in temperatures from Magdalenian times up to the Holocene is clearly recorded also in the sequences of Santimamiñe^3^, El Mirón^6^, and Kiputz IX^2^.

Along HE 3 and HE 2, contrary to the rest of the proxies, the small mammals do not display particularly open landscapes. At the top of the Upper Solutrean, there are peaks of forest according to the small-vertebrate evidence (especially amphibians and reptiles) during the LGM, while the stable isotopes of red deer and the marine signals suggest priority of meadows and low temperatures. Finally, towards Epipaleolithic times, there is a retreat of woodlands after the small vertebrates, what is not coherent with what is inferred from the marine record. It should be stated then that together with coincidences, our sequence displays also some discrepancies: either some proxies do not reflect specific events with the same intensity as others, or they show opposite trends in few cases. These inconsistencies represent a challenge for future studies.

## Methods

### Small-vertebrate materials

The assemblage includes nearly 31400 elements, of which 2470 were identified either to the family, genus and/or species level. To obtain the samples, the sediment from the different stratigraphic levels of the cave was water-screened using a stack of sieves of decreasing mesh size (4 and 0.5 mm). The small vertebrates were collected from residue coarser than 0.5 mm. Fossils were sorted, classified, counted, and studied with the aid of a binocular microscope (Nikon SMZ-U; 7x, 20x, and 40x magnifications). Most of the elements are teeth, isolated mandibles, skull fragments, and postcranial bones.

### Systematic attribution and quantification

**Figure 2:**
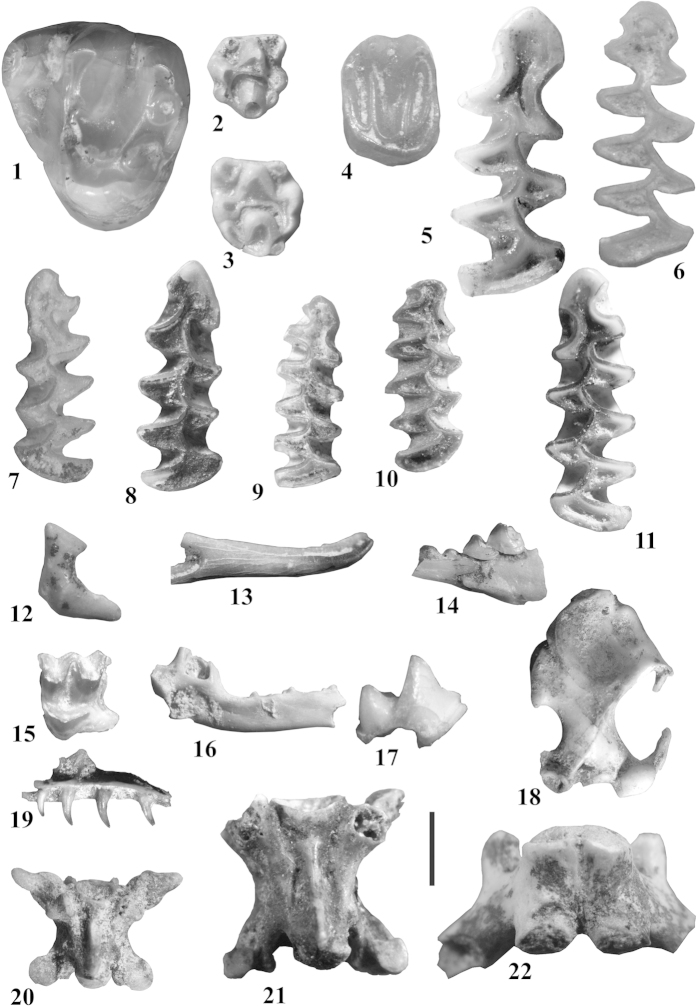
Selected specimens of small vertebrates from Antoliñako Koba (Gautegiz-Arteaga, Bizkaia, Spain). *Marmota marmota* (1) right D4 in occlusal view; *Apodemus sylvaticus* (2) right M2 in occlusal view; *A. flavicollis* (3) left M2 in occlusal view; *Eliomys quercinus* (4) right M1 or M2 in occlusal view; *Arvicola amphibius* (5) right m1 in occlusal view; *Pliomys lenki* (6) right m1 in occlusal view; *Microtus* (*Alexandromys*) *oeconomus* (7) left m1 in occlusal view; *Chionomys nivalis* (8) right m1 in occlusal view; *Microtus* (*Terricola*) *lusitanicus* (9) right m1 in occlusal view; *M.* (*Microtus*) *agrestis* (10) right m1 in occlusal view; *M.* (*M.*) *arvalis* (11) left m1 in occlusal view; Neomyni indet. (12) left mandibular condyle in posterior view; *Neomys* sp. (13) right i1 in lateral view; *Sorex* cf. *coronatus* (14) incomplete left mandible with i1 (broken), a1 and p4; (15) left M1 in occlusal view; *S. minutus* (16) incomplete left mandible in medial view; *Crocidura russula* (17) right m1 in lateral view; *Talpa* sp. (18) left humerus in anterior view; *Anguis fragilis* (19) incomplete right maxillae in medial view; *Coronella girondica* (20) trunk vertebrae in ventral view; *Vipera* sp. (21) trunk vertebrae in ventral view; *Rana* gr. *temporaria-iberica* (22) sacral vertebrae in ventral view. **Scale bar =** 1 mm for figures 2–11, 13, 16 and 17; 2 mm for figures 1, 12, 14, 15, and 19–22; 4 mm for figure 18.

**Figure 4:**
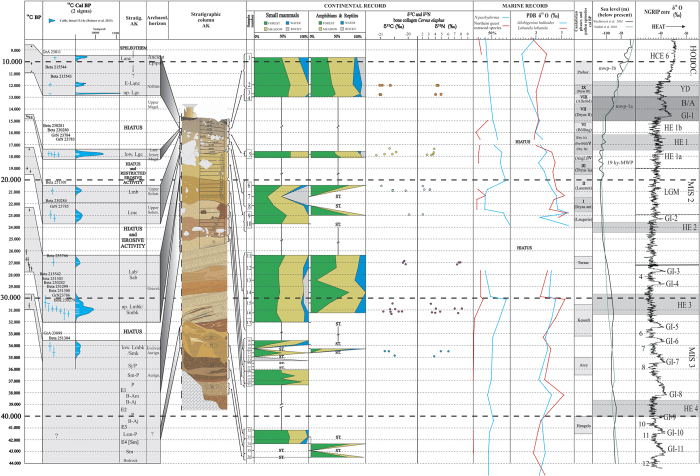
Multi-proxy estimated palaeoenvironmental reconstruction for the Cantabrian Region during the Upper Pleistocene. From left to right, columns represent the ^14^C BP radiocarbon dates (with laboratory codes); the ^14^C BP 2σ calibrated (cal) radiocarbon dates using the IntCal13.14c data set^66^; the stratigraphic levels of AK; the cultural horizons defined by their archaeological contents; the stratigraphic sequence of AK; the location of the samples; the small-mammal taxa grouped by their habitat requirements; the amphibian and reptile taxa grouped by their habitat requirements; the δ^13^C and δ^15^N values of *C. elaphus* from AK; the relative abundances of *N. pachyderma* sin (planktonic foraminifer) and “Northern guest” species (benthic ostracods) over time; the δ^18^O signals of *G. bulloides* (planktonic foraminifer) and *L. lobatula* (benthic foraminifer) over time; the sedimentological^41^ and palynological^42^ episodes for the Cantabrian Region; the North-Atlantic sea-level curves^43^,^44^; and a δ^18^O curve obtained from a deep ice core of North Greenland (NGRIP^45^) displaying some well-known North-Atlantic climatic episodes (HE 1 phases^52^ and limits between MIS^68^ were taken from elsewhere). **AK**, Antoliñako Koba; **ST.**, sterile layer; **Prebor.**, Preboreal; **MWP**, melt water pulse; **HCE**, Holocene Cooling Event; **YD**, Younger Dryas; **B/A**, Bölling/Alleröd; **GI**, Greenland Interstadial; **HE**, Heinrich Event; **LGM**, Last Glacial Maximum; **MIS**, Marine Isotope Stages.

Each small-vertebrate taxon was identified based on cranial and post-cranial diagnostic elements: isolated teeth for the Murinae and Gliridae; first lower molars for the Arvicolinae; mandibles, maxillae, isolated teeth, and post-cranial skeleton for the Talpidae and Soricidae; humerus, ilium, scapula, and sacrum for amphibians; skull elements, vertebrae and osteoderms for lizards, and trunk vertebrae for snakes. The taxonomic classification follows well-known references^58^,^59^. In spite that in Fig. 2 we show two discernible second upper molars of *Apodemus sylvaticus* and *A. flavicollis* (Fig. 2(2, respectively), most elements of the sample exhibit ambiguous morphologies. The relative ratios of fossil species were established with the minimum number of individuals (MNI), also used as a quantitative measure to reconstruct the palaeoenvironment. To determine the MNI, a diagnostic tooth (e.g., first lower molar in arvicolines) or post-cranial elements were considered, taking into account laterality and sex whenever possible.

### Taphonomic remarks

The light to moderate gastric digestion and scant breakage observed in the small-vertebrate remains, indicates that the bones were likely accumulated by an avian predator of category 1^5^ such as a barn owl (*Tyto alba*), which is an opportunistic rather than a selective hunter. However, there are certain instances of great to extreme modification, which means categories IV to V on Andrews' scale^5^. In these cases, the agents of deposition were most probably small-mammalian carnivores. The pattern of skeletal-element frequency for the slow worm, with lack of digestion traces, would correspond to *in situ* mortality. Therefore, there are no signs of alteration suggesting that the Antoliña assemblage is not representative of the ecosystem in the immediate vicinity of the cave at the time when the remains were deposited.

### Habitat weightings

We distribute each small-vertebrate taxon in the habitat(s) where it is possible to find them at present, especially in the Cantabrian Region^8^,^60^,^61^. Habitats were divided into four types^3^,^6^,^7^,^9^,^10^,^11^, which are detailed as follows (see supplementary Tables 1 and 2): *Forest*: mature woodland, including woodland margins and forest patches, with moderate ground cover; *Meadow*: evergreen open areas with dense pastures and suitable topsoil; *Water*: streams, lakes, ponds, and marshes; *Rocky*: areas with suitable rocky or stony substratum. The Meadow type, as defined here, is particularly suitable for the well-known humid conditions of the Cantabrian Range^60^,^61^. For the rest of Mediterranean Iberia, a Grassland or Open-dry category is required.

### Stable carbon and nitrogen isotope analysis

A total of 38 samples from AK specimens were analyzed, ten of which were not considered for interpretations due to low concentrations of collagen (supplementary Table 3). Only 28 samples had C/N atomic ratios between 2.9 and 3.6, which indicates good collagen preservation^62^. The stratigraphic distribution of the useful samples is as follows: evolved Aurignacian (5), Gravettian (12), Upper Solutrean (3), Lower Magdalenian (6), and Azilian (5). Bone collagen (coll) from red deer (*C. elaphus*) long bones was extracted following a specific procedure^63^. Three-hundred mg of bone sample powder was demineralized in 1 M HCl for 20 min at ambient temperature until all minerals had dissolved. Samples were then rinsed with distilled water and 0.125 M NaOH was added to remove humic acid. They were then rinsed with distilled water again and gelatinized in a pH 3 HCl solution for 17 h at 90 °C. The filtered supernatant containing the soluble collagen was then collected, frozen, and lyophilized. Collagen (2.5–3.5 mg) was loaded into a tin capsule for continuous flow combustion and isotopic analysis. Isotope analyses were performed for carbon and nitrogen isotopes using a continuous-flow isotope ratio mass spectrometer (EA-IRMS) at Iso-Analytical (Cheshire, UK). The bone collagen amount is 12.8–0.36 %wt. The C_coll_ and N_coll_ contents are above 25.6% and 8.2% wt respectively, and the C/N atomic ratio is 3.2–3.6, which corresponds to well-preserved collagen^63^. Multiple samples of the liver standard NBS-1577B and ammonium sulphate IA-R045 working standard were run to confirm instrument accuracy. Replicate analysis of the NBS-1577B δ^13^C standard during runs gave a ^13^C/^12^C of **–**21.65 **±** 0.07 (s, n = 12); NBS-1577B δ^15^N standard during runs gave a ^15^N/^14^N of 7.63 **±** 0.14 (s, n = 12), and the IA-R045 working standard during runs gave a ^15^N/^14^N of **−**4.70 **±** 0.05 (s, n = 5).

### Stable oxygen isotope analysis

Values of δ^18^O were measured on the shells of the planktonic foraminifer species *Globigerina bulloides*, and of the benthic foraminifer species *Lobatula lobatula* (supplementary Table 4). Between 2 and 18 specimens per sample were handpicked for each species. These individuals were washed in alcohol and placed in an ultrasonic cleaner for less than 10 seconds in order to eliminate any contaminating residual adhering to the foraminifer test. δ^18^O data obtained are reported referred to the PeeDee belemnite (V-PDB) standard. Analyses were accomplished in the Leibniz Laboratory for Radiometric Dating and Stable Isotope Research (Kiel University, Germany) using a “Finnigan DELTAplusXL” mass-spectrometer coupled to a “GasBench II” continuous flow interface, equipped with a “CTC Combi PAL Autosampler”. The analytical error of analysis was lower than ±0.1‰.

### Marine record

It is based on a composite stack of the faunistic (foraminifers and ostracods) and isotopic (δ^18^O) analysis of two cores from the outer Basque shelf (Southern Bay of Biscay): KS04-16 (43°32'66 N latitude, 2°05'72 W longitude, 294 m water depth), taken at the eastern flank of the San Sebastian Canyon, and KS05-05 (43°30'597 N latitude, 2°13'76 W longitude, 259 mwd.), obtained at the western flank (see Fig. 1b for location, and supplementary Table 4 for raw data).

The stratigraphic framework of both cores has been proposed elsewhere^4^,^28^. It is based on a combination of calibrated AMS ^14^C dates and an independent local event-stratigraphy constructed tuning the percentage of *N. pachyderma* left coiling (sin) to the NGRIP ice core δ^18^O record from Greenland (with the GICC05 time-scale)^45^, and to the MD95-2042 marine sedimentary core δ^18^O record from the SW Iberian Peninsula shelf^35^. The section studied here covers ~35 ka (from 43.1 ka cal BP to 7.9 ka cal BP), with the loss of 3.8 ka during the beginning of MIS 2 (27.7–23.9 ka cal BP), and of 0.6 ka during HE 1 (17.1–16.5 ka cal BP) due to two erosive hiatuses. Sedimentation rates range between 2 and 10 cm/ka resulting in a time resolution of approximately 1400 years for MIS 3 interval, ~620 years for MIS 2, and ~2200 years for the beginning of MIS 1.

In order to study the benthic and planktonic faunas, 11 samples (continuous intervals of 5 cm) were analyzed from core KS05-05, and 18 samples (intervals between 2 cm and 13 cm) from core KS04-16. Samples were water-screened with a 150 μm mesh sieve. All the ostracods present in the samples and at least 300 individuals of planktonic foraminifers per sample were picked^4^. Several taxonomical references have been used to identify the ostracod and foraminifer species^49^,^64^,^65^. To identify the input of cold circumpolar surface and bottom water masses into the Basque shelf, only the percentage of polar planktonic foraminifer species *N. pachyderma* sin, and the accumulative percentage of “Northern guest” ostracod species, have been considered in this work.

## Additional Information

**How to cite this article**: Rofes, J. *et al.* Combining Small-Vertebrate, Marine and Stable-Isotope Data to Reconstruct Past Environments *Sci. Rep.* **5**, 14219; doi: 10.1038/srep14219 (2015).

## Supplementary Material

Supplementary Information

## Figures and Tables

**Figure 3:**
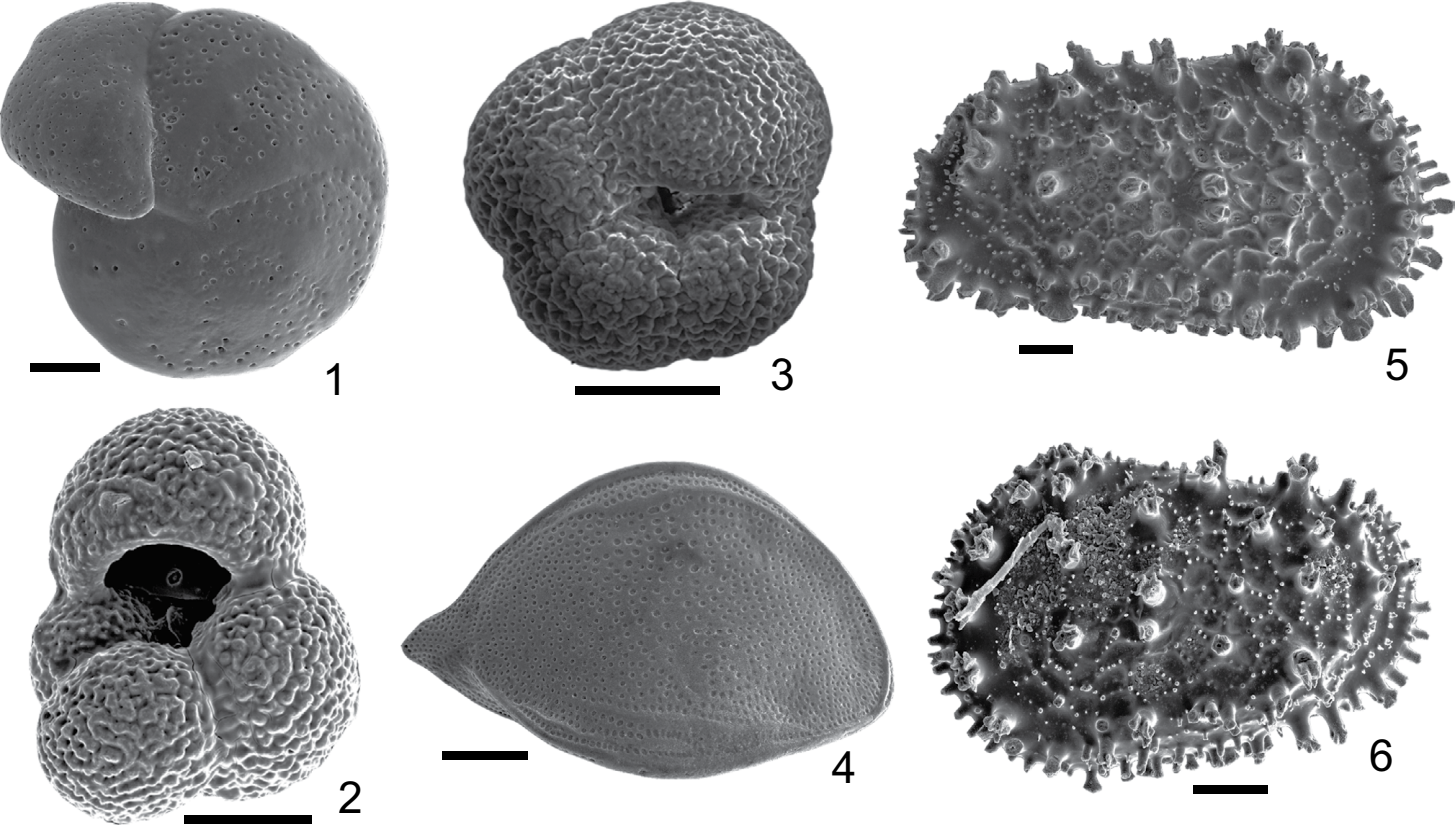
Selected specimens of foraminifers and ostracods from the outer Basque shelf (Southern Bay of Biscay) used in this study. (1) Benthic foraminifer species: *Lobatula lobatula*, spiral view. (2-3) Planktonic foraminifer species: (2) *Globigerina bulloides*, oral view; (3) *Neogloboquadrina pachyderma* left coiling (sin), oral view. (4–6) “Northern guest” ostracod species (all lateral external views): (4) *Cytheropteron testudo*, right valve; (5) *Acanthocythereis dunelmensis*, right valve; (6) “*Trachyleberis*” sp., left valve. Scale bar = 100 μm.

**Table 1:** List of radiocarbon ages from the Antoliñako Koba stratigraphic column.

**Cultural horizon**	**Level**	**Radiocarbon age**			**Lab code**	**Sample**
		^**14**^**C yr BP**	**Cal yr BP 2σ**	**Mean prob.**		
Epipaleolithic	Lanc	8680 ± 60	9850–9490	9642	GrA-23811	Charcoal
Azilian	E-Lanc	10220 ± 40	12140–11740	11935	Beta-215544	Bone (AK-3)
Azilian	Upper Lgc	10800 ± 40	12800–12680	12712	Beta-215543	Bone (AK-2)
late Lower Magdalenian	Lower Lgc	14580 ± 70 – 14680 ± 100	17870–17710/17930–17730	17760–17863	Beta-230281/GrN-23784	Bone (AK-10)/Bone
Upper Solutrean	Lmb	17340 ± 100	21090–20530	20918	Beta-251301	Bone (AK-21)
Upper Solutrean	Lmc	19020 ± 120	23250–22570	22897	Beta-230284	Bone (AK-13)
Upper Solutrean	Lmc	19280 ± 120	23450–22850	23225	GrN-23785	Bone
Gravettian	Lab/Sab	22640 ± 120	28000–26760	26973	Beta-233766	Bone (AK-14)
Gravettian	Upper Lmbk/Smbk	26710 ± 180	31880–31280	30905	Beta-230282	Bone (AK-11)
Gravettian	Upper Lmbk/Smbk	27520 ± 190	32430–31750	31326	Beta-230279	Bone (AK-7)
Evol. Aurignacian	Lower Lmbk/SmK	29990 ± 230	34670–33870	34065	GrA-23898	Charcoal
Evol. Aurignacian	Lower Lmbk/SmK	30640 ± 240	35290–34210	34576	Beta-251304	Bone (AK-31)

Including cultural horizons, chrono-stratigraphic units, laboratory codes and the elements from where the samples were taken. Dates were calibrated at 95% confidence intervals (2σ) using the IntCal13.14c data set^67^. Mean probability calculated with the Calib7.0.4^68^.
