# Supplementary material for: Combining Small-Vertebrate, Marine and Stable-Isotope Data to Reconstruct Past Environments
Source: Sci Rep. 2015 Sep 22;5:14219. doi: 10.1038/srep14219 (PMC4585735; doi:10.1038/srep14219)
Supplement: Supplementary Information [file srep14219-s1.pdf]

# COMBINING SMALL-VERTEBRATE, MARINE AND STABLE ISOTOPE DATA TO RECONSTRUCT PAST ENVIRONMENTS

Juan Rofes*, Naroa Garcia-Ibaibarriaga, Mikel Aguirre, Blanca Martínez-García, Luis Ortega, María Cruz Zuluaga, Salvador Bailon, Ainhoa Alonso-Olazabal, Jone Castaños & Xabier Murelaga

**Supplementary Table 1. Number of identified specimens (NISP) and minimum number of individuals (MNI) of the small-mammal taxa from Antoliñako Koba.** Values organized by cultural horizons and chrono-stratigraphic units. **Right:** Habitat weightings.

| Cultural Horizon | Anc. Epipal. | | Azilian | | * | | Upper Solutrean | | | | Gravettian | | | | Evolved Aurig. | | | | Aurignacian | | | | | | ? | | | | Habitat distribution | | | |
|---|---|---|---|---|---|---|---|---|---|---|---|---|---|---|---|---|---|---|---|---|---|---|---|---|---|---|---|---|---|---|---|---|
| **Level** | Lanc | | up. Lgc | | low. Lgc | | Lmb | | Lmc | | Lab/Sab | | ** | | low Lmbk/Smk | | | | Sj/P | | Sm-P | | P | | Lsm-P | | Sm | | | | | |
| **Sample** | 1-2 | | 3-4 | | 5 | | 6-8 | | 9-10 | | 11-13 | | 14-17 | | 18-19 | | 21-23 | | 26 | | 27-30 | | 31 | | 32-33 | | 34 | | Fo | Me | Wa | Ro |
| | NISP | MNI | NISP | MNI | NISP | MNI | NISP | MNI | NISP | MNI | NISP | MNI | NISP | MNI | NISP | MNI | NISP | MNI | NISP | MNI | NISP | MNI | NISP | MNI | NISP | MNI | NISP | MNI | | | | |
| *Talpa* sp. | 6 | 2 | 1 | 1 | 4 | 1 | 4 | 2 | 1 | 1 | 24 | 4 | 33 | 5 | 1 | 1 | 1 | 1 | | | 2 | 1 | | | 1 | 1 | 1 | 1 | 0.5 | 0.5 | | |
| *Sorex* cf. *coronaus* | 4 | 2 | 5 | 2 | | | 5 | 1 | 4 | 1 | 14 | 4 | 29 | 7 | 6 | 2 | 2 | 1 | | | 2 | 2 | | | 12 | 2 | | | 0.5 | 0.5 | | |
| *Sorex minutus* | 1 | 1 | | | | | | | | | | | | | | | | | | | | | | | 1 | 1 | 1 | 1 | 0.5 | 0.5 | | |
| *Neomys* sp. | 2 | 1 | | | | | | | | | | | | | | | | | | | | | | | | | 2 | 1 | | | 1 | |
| *Crocidura russula* | 1 | 1 | | | | | | | | | | | | | | | | | | | | | | | | | | | 0.5 | 0.5 | | |
| *Arvicola amphibius* | 1 | 1 | 4 | 3 | | | 3 | 2 | | | 4 | 2 | 4 | 3 | 2 | 1 | | | | | | | | | 2 | 2 | 5 | 3 | 0.5 | 0.5 | | |
| *Chionomys nivalis* | | | | | | | 1 | 1 | | | 1 | 1 | 2 | 2 | | | 1 | 1 | | | | | | | | | | | | | | 1 |
| *Microtus (Terricola) lusitanicus* | 1 | 1 | 1 | 1 | 2 | 1 | | | 1 | 1 | 2 | 2 | 2 | 2 | | | | | 1 | 1 | | | 1 | 1 | 6 | 4 | 1 | 1 | 0.5 | 0.5 | | |
| *Microtus (Alexandromys) oeconomus* | 2 | 2 | | | 2 | 1 | 1 | 1 | 2 | 2 | 6 | 4 | 4 | 2 | | | | | | | | | | | 6 | 3 | | | 0,5 | 0.5 | | |
| *Microtus (Microtus) arvalis* | 1 | 1 | 2 | 1 | 1 | 1 | 7 | 3 | | | 17 | 9 | 17 | 10 | 2 | 1 | 1 | 1 | | | 1 | 1 | | | 14 | 11 | 6 | 3 | 0.5 | 0.5 | | |
| *Microtus (Microtus) agrestis* | | | | | | | 1 | 1 | | | | | 6 | 4 | | | | | | | | | | | 3 | 2 | | | 0.2 | 0.8 | | |
| *Pliomys lenki* | | | | | | | | | | | | | | | | | | | | | 2 | 2 | | | 9 | 6 | 5 | 4 | 1 | | | |
| *Apodemus* gr. *sylvaticus-flavicollis* | 23 | 5 | 13 | 4 | 6 | 2 | 2 | 1 | 5 | 1 | 3 | 2 | 6 | 4 | 3 | 2 | | | | | 4 | 2 | | | 34 | 8 | 19 | 6 | 1 | | | |
| *Glis glis* | | | | | | | | | | | | | | | | | | | | | | | | | 2 | 1 | | | 1 | | | |
| *Eliomys quercinus* | | | | | | | | | | | | | | | | | | | | | | | | | | | 1 | 1 | 0.8 | 0.2 | | |
| *Marmota marmota* | | | | | | | | | | | | | | | | | | | | | | | | | | | 1 | 1 | | | | 1 |
| **Totals** | 42 | 17 | 26 | 12 | 15 | 6 | 24 | 12 | 13 | 6 | 71 | 28 | 103 | 39 | 14 | 7 | 5 | 4 | 1 | 1 | 11 | 8 | 1 | 1 | 90 | 41 | 42 | 22 | | | | |
| **Nº of species** | 7 | | 3 | | 5 | | 4 | | 5 | | 6 | | 7 | | 4 | | 3 | | 1 | | 1 | | 1 | | 6 | | 10 | | | | | |

*late Lower Magdalenian; **upper Lmbk/Smbk. Fo, Forest; Me, Meadow; Wa, Water; Ro, Rocky.

**Supplementary Table 2. Number of identified specimens (NISP) and minimum number of individuals (MNI) of the amphibian and reptile taxa from Antoliñako Koba.** Values organized by cultural horizon and chrono-stratigraphic units. **Right:** Habitat weightings.

| **Cultural Horizon** | Anc. Epipal. | | Azilian | | * | | Upper Solutrean | | | | Gravettian | | | | Evol. Aurig. | | ? | | | | **Habitat distribution** | | | |
|---|---|---|---|---|---|---|---|---|---|---|---|---|---|---|---|---|---|---|---|---|---|---|---|---|
| **Level** | Lanc | | up. Lgc | | low. Lgc | | Lmb | | Lmc | | Lab/Sab | | ** | | l. Lmbk/Smk | | Sm | | | | | | | |
| **Sample** | 1-2 | | 3-4 | | 5 | | 6 | | 9 | | 11-13 | | 14-17 | | 20 | | 34 | | 36 | | Fo | Me | Wa | Ro |
| | NISP | MNI | NISP | MNI | NISP | MNI | NISP | MNI | NISP | MNI | NISP | MNI | NISP | MNI | NISP | MNI | NISP | MNI | NISP | MNI | | | | |
| *Alytes obstetricans* | | | | | | | | | | | 2 | 1 | | | | | | | | | 0.5 | 0.3 | 0.2 | |
| *Bufo bufo* | 1 | 1 | | | | | | | | | 7 | 3 | 1 | 1 | 1 | 1 | | | | | 0.2 | 0.6 | 0.2 | |
| *Bufo calamita* | | | | | | | | | | | 1 | 1 | 2 | 2 | | | | | | | | 0.8 | 0.2 | |
| *Rana gr. temporaria-iberica* | 2 | 2 | 1 | 1 | | | 2 | 1 | | | 22 | 6 | 29 | 7 | | | 2 | 2 | | | 0.5 | 0.5 | | |
| *Triturus* sp. | 1 | 1 | 1 | 1 | | | | | | | | | | | | | | | | | | 0.5 | 0.5 | |
| *Lacertidae* | 2 | 1 | | | | | | | | | | | 1 | 1 | | | 1 | 1 | | | | | | |
| *Anguis fragilis* | 2450 | 2 | 4504 | 2 | 1474 | 1 | 201 | 1 | 207 | 1 | 16 | 2 | 14 | 2 | | | 2 | 1 | 5 | 1 | 0.7 | 0.3 | | |
| *Chalcides striatus* | 3 | 2 | | | | | | | | | | | | | | | | | | | 0.2 | 0.5 | | 0.3 |
| *Coronella girondica* | 3 | 2 | 3 | 2 | 2 | 1 | | | | | | | | | | | | | | | 0.2 | 0.6 | | 0.2 |
| cf. *Natrix* | 1 | 1 | | | | | | | | | | | | | | | | | | | | | | |
| *Vipera* sp. | | | | | | | | | | | 1 | 1 | | | | | | | | | 0.3 | 0.5 | | 0.3 |
| **Totals** | 2463 | 12 | 4509 | 6 | 1476 | 2 | 203 | 2 | 207 | 1 | 49 | 14 | 47 | 13 | 1 | 1 | 5 | 4 | 5 | 1 | | | | |
| **Nº of species** | 8 | | 4 | | 2 | | 2 | | 1 | | 6 | | 5 | | 1 | | 3 | | 1 | | | | | |

*late Lower Magdalenian; **upper Lmbk/Smbk. Fo: Forest; Me, Meadow; Wa, Water; Ro, Rocky.

**Supplementary Table 3. Results of the isotopic analysis ($\delta^{15}N$ and $\delta^{13}C$) of collagen of red deer bones from Antoliñako Koba.** Values organized by cultural horizons and chrono-stratigraphic units. Underlined bold values at the C/N ratio column were left aside from interpretations due to low concentrations of collagen. The levels other than those shown in Fig. 4 correspond to locally restricted facies of the main stratigraphic units.

| Cultural Horizon | Level | Sample ID | $\delta^{15}N$ | $\delta^{13}C$ | C/N |
|---|---|---|---|---|---|
| Azilian | E1 (Lanc) | AK-05 | 4.45 | -20.89 | 3.4 |
| Azilian | E1 (Lanc) | AK-05A | 4.39 | -21.04 | 3.45 |
| Azilian | E1 (Lanc) | AK-03 | 3.94 | -21.44 | **4.07** |
| Azilian | E1 (Lanc) | AK-03A | 3.81 | -20.72 | 3.39 |
| Azilian | Up. Lgc | AK-02 | 2.71 | -22.14 | **5.52** |
| Azilian | Up. Lgc | AK-2A | 4.05 | -21.03 | 3.33 |
| Azilian | Up. Lgc | AK-2B | 4.93 | -20.96 | 3.46 |
| Lower Magdalenian | Low. Lgc | AK-36 | 3.88 | -20.71 | 3.41 |
| Lower Magdalenian | Low. Lgc (lamc) | AK-08 | 3.71 | -20.58 | 3.39 |
| Lower Magdalenian | Low. Lgc (lamc) | AK-09 | 3.46 | -20.84 | 3.35 |
| Lower Magdalenian | Low. Lgc | AK-35 | 3.81 | -21.76 | **3.8** |
| Lower Magdalenian | Low. Lamc | AK-10 | 3.79 | -20.40 | 3.29 |
| Lower Magdalenian | Low. Lgc | AK-06 | 2.83 | -21.13 | 3.33 |
| Lower Magdalenian | Lzg | AK-04 | 2.15 | -20.96 | 3.25 |
| Upper Solutrean | Lmb | AK-20 | 3.24 | -20.55 | 3.41 |
| Upper Solutrean | Lmb | AK-21 | 2.37 | -20.10 | 3.26 |
| Upper Solutrean | H3-Lmc | AK-13 | 2.18 | -19.96 | 3.3 |
| Gravettian | Lab | AK-14 ext | 7.84 | -20.17 | 3.34 |
| Gravettian | | AK-14 int | 7.66 | -20.21 | 3.3 |
| Gravettian | Lab (roof) | AK-12 | 7.43 | -20.13 | 3.38 |
| Gravettian | Lmb | AK-01 | 4.00 | -20.47 | 3.5 |
| Gravettian | Lab | AK-07 | 4.92 | -20.61 | 3.31 |
| Gravettian | Up. Lmbk | AK-11 | 8.12 | -20.46 | 3.34 |
| Gravettian | Lmcb | AK-18 | 4.01 | -20.66 | 3.42 |
| Gravettian | Lmcb | AK-19 | 4.83 | -20.90 | 3.29 |
| Gravettian | Lmck | AK-17 | 7.53 | -20.23 | 3.38 |
| Gravettian | Lmck | AK-15 | 4.08 | -20.70 | 3.45 |
| Gravettian | Lmck | AK-16 | 7.05 | -20.32 | 3.4 |
| Gravettian | Up. Smbk | AK-30 | 3.36 | -20.58 | 3.56 |
| Evol. Aurignacian | Smb+Sm | AK-31 | 6.02 | -20.70 | 3.5 |
| Evol. Aurignacian | Lsrb | AK-26 | 6.14 | -21.38 | **3.81** |
| Evol. Aurignacian | Lsrb | AK-28 | 6.63 | -21.51 | **4.26** |
| Evol. Aurignacian | Lsrb | AK-29 | 4.88 | -20.86 | 3.54 |
| Evol. Aurignacian | Lsrb | AK-23 | 3.89 | -20.47 | **3.78** |
| Aurignacian | Sr-E | AK-37 | 3.45 | -22.73 | **5.19** |
| Aurignacian | Sr-E | AK-39 | 3.28 | -21.90 | **3.73** |
| Aurignacian | P+Sm-P | AK-33 | 3.49 | -22.40 | **4.39** |
| Aurignacian | P+Sm-P | AK-34 | 3.62 | -21.81 | **4.14** |

**Supplementary Figure 1. Boxplot of $\delta^{13}C$ (a) and $\delta^{15}N$ (b) collagen values for red deer bones from Antoliñako Koba.** Ranges distributed by cultural horizons.

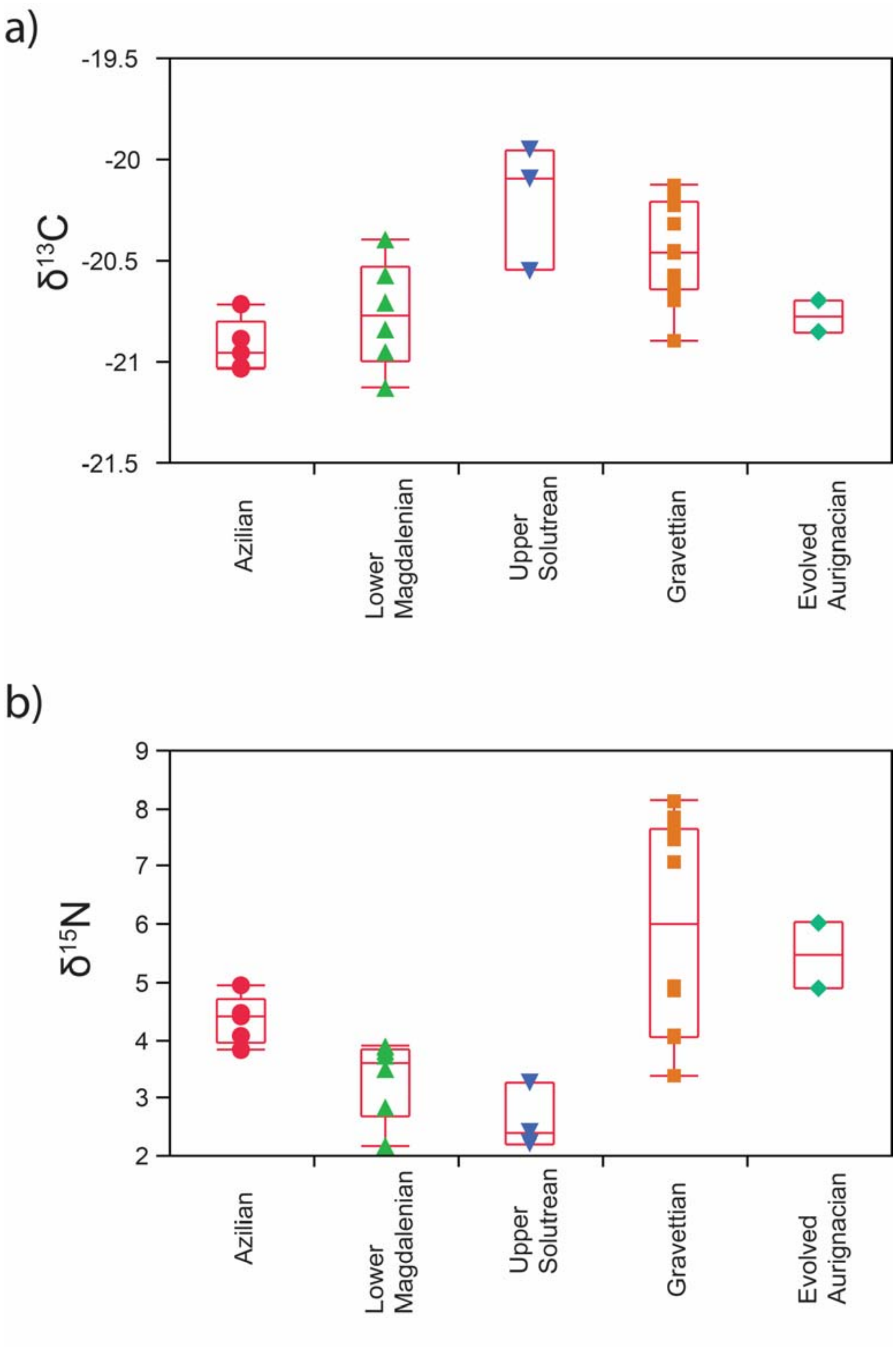

**Supplementary Table 4. Composite marine record of cores KS04-16 and KS05-05 (Basque shelf).** Estimated age is based on the stratigraphic framework explained in the Methods chapter.

| Estimated age (years BP) | *Neogloboquadrina pachyderma* sin. (%) | "Northern guest" ostracod species (%) | $\delta^{18}O$ (PDB) *Lobatula lobatula* | $\delta^{18}O$ (PDB) *Globigerina bulloides* |
|---|---|---|---|---|
| 7887 | 33.64 | 14.29 | 1.826 | 1.986 |
| 9356 | 36.39 | 22.22 | 2.423 | 1.452 |
| 12539 | 40.43 | 0 | 2.062 | 1.676 |
| 14988 | 53.55 | 38.64 | 2.098 | 2.144 |
| 15969 | 61.94 | 9.68 | 1.971 | 1.976 |
| 16530 | 60.49 | 18.18 | 1.930 | 2.262 |
| 17090 | 56.97 | 17.78 | 2.146 | 2.279 |
| 17651 | 70.68 | 15 | 2.623 | 2.427 |
| 18554 | 70.23 | 14.29 | 2.747 | 2.096 |
| 19306 | 78.62 | 0 | 2.658 | 2.330 |
| 20058 | 88.01 | 0 | 2.722 | 2.477 |
| 20810 | 36.48 | 13.33 | 2.806 | 2.628 |
| 21313 | 32.04 | 33.33 | 3.121 | 2.804 |
| 21648 | 33.22 | 10 | 2.710 | 2.786 |
| 22485 | 31.17 | 10 | 2.527 | 2.854 |
| 22779 | 82.22 | 0 | 3.198 | 3.274 |
| 23170 | 86.26 | 0 | 2.889 | 2.163 |
| 23855 | 90.94 | 0 | - | 3.262 |
| 27700 | 30.22 | 14.95 | 2.452 | 2.602 |
| 29871 | 46.39 | 22.05 | 2.169 | 2.509 |
| 30100 | 82.91 | 26.73 | 3.130 | 2.767 |
| 31708 | 73.16 | 3.93 | 2.760 | 2.618 |
| 33316 | 52.37 | 1.23 | 2.280 | 2.374 |
| 34293 | 51.52 | 1.54 | 2.582 | 2.457 |
| 36898 | 48.46 | 0.26 | 2.339 | 2.164 |
| 38200 | 38.59 | 0 | 3.087 | 2.606 |
| 40373 | 54.84 | 0 | 2.188 | 2.226 |
| 41300 | 40.45 | 0.24 | 2.181 | 2.269 |
| 43133 | 41.1 | 0.92 | 1.899 | 2.364 |
| 44967 | 41.23 | 0 | 2.355 | 2.144 |

"Northern guest" ostracod species referred as accumulative % of *A. dunelmensis, C. testudo* and “*Trachyleberis*” sp.
